# Abrupt Photoperiod Changes Differentially Modulate Hepatic Antioxidant Response in Healthy and Obese Rats: Effects of Grape Seed Proanthocyanidin Extract (GSPE)

**DOI:** 10.3390/ijms242317057

**Published:** 2023-12-02

**Authors:** Antonio J. Cortés-Espinar, Néstor Ibarz-Blanch, Jorge R. Soliz-Rueda, Enrique Calvo, Francisca Isabel Bravo, Miquel Mulero, Javier Ávila-Román

**Affiliations:** 1 Nutrigenomics Research Group, Department of Biochemistry and Biotechnology, Universitat Rovira i Virgili, 43007 Tarragona, Spain; antoniojesus.cortes@estudiants.urv.cat (A.J.C.-E.); nestor.ibarz@urv.cat (N.I.-B.); jorgericardo.soliz@urv.cat (J.R.S.-R.); enrique.calvo@urv.cat (E.C.); franciscaisabel.bravo@urv.cat (F.I.B.); 2 Nutrigenomics Research Group, Institut d’Investigació Sanitària Pere Virgili, 43007 Tarragona, Spain; 3 Molecular and Applied Pharmacology Group (FARMOLAP), Department of Pharmacology, Universidad de Sevilla, 41012 Sevilla, Spain

**Keywords:** oxidative stress, phenolic compounds, obesity, GSPE, chronodisruption, liver, *zeitgeber*, chronotherapy

## Abstract

Disruptions of the light/dark cycle and unhealthy diets can promote misalignment of biological rhythms and metabolic alterations, ultimately leading to an oxidative stress condition. Grape seed proanthocyanidin extract (GSPE), which possesses antioxidant properties, has demonstrated its beneficial effects in metabolic-associated diseases and its potential role in modulating circadian disruptions. Therefore, this study aimed to assess the impact of GSPE administration on the liver oxidant system of healthy and diet-induced obese rats undergoing a sudden photoperiod shift. To this end, forty-eight photoperiod-sensitive Fischer 344/IcoCrl rats were fed either a standard (STD) or a cafeteria diet (CAF) for 6 weeks. A week before euthanizing, rats were abruptly transferred from a standard photoperiod of 12 h of light/day (L12) to either a short (6 h light/day, L6) or a long photoperiod (18 h light/day, L18) while receiving a daily oral dose of vehicle (VH) or GSPE (25 mg/kg). Alterations in body weight gain, serum and liver biochemical parameters, antioxidant gene and protein expression, and antioxidant metabolites were observed. Interestingly, GSPE partially ameliorated these effects by reducing the oxidative stress status in L6 through an increase in *GPx1* expression and in hepatic antioxidant metabolites and in L18 by increasing the NRF2/KEAP1/ARE pathway, thereby showing potential in the treatment of circadian-related disorders by increasing the hepatic antioxidant response in a photoperiod-dependent manner.

## 1. Introduction

Organisms have developed biological rhythms to adapt to their environment, resulting in increased energy efficiency as physiological, metabolic, and behavioral processes coordinate with external cues known as *zeitgebers* (ZTs) [1]. These rhythms appeared as a response to the Earth’s rotational movement (i.e., circadian rhythms) and the translational movement (i.e., circannual rhythms) that determine days and seasons, respectively [2]. As a result, processes such as blood pressure, sleep/wake cycles, or liver metabolism are modulated by circadian rhythms, with a rhythmicity of approximately 24 h [3]. The main external cue that entrains biological rhythms is the light, which in mammals reaches the suprachiasmatic nucleus (SCN) of the hypothalamus. This central pacemaker recognizes both the light and the hours of daylight during this 24 h period (i.e., photoperiod) allowing the organism to adapt to the time of day and year by sending signals to entrain the peripheral oscillators present in virtually all tissues, including skeletal muscle, liver, or adipose tissue [4,5]. Subsequently, these oscillators regulate the expression of tissue-specific genes and affect cellular functions. Several harmful effects of altering the light/dark cycle have been described in this context, including increased body weight gain, alterations in lipid metabolism, impacts on bone health, colon physiology, and even mental disorders [6,7,8,9,10].

Furthermore, exercise, temperature, or food intake can also entrain biological clocks. In fact, the main ZT that entrains the peripheral oscillator is the feeding pattern, including not only the feeding schedule but also the diet composition [11]. In this context, the effects of a high-fat diet on circadian clock machinery consist of altering the expression of clock genes in peripheral tissues but not in the SCN [12]. In fact, Pendergast and colleagues described a 5 h phase advance in the liver clock of mice compared to the SCN after a week of a high-fat diet, promoting the misalignment between the central and the peripheral clocks [13]. Therefore, current lifestyles, including irregular eating schedules or high calorie intake, exposure to artificial light, shift work, and jet lag, can lead to chronodisruption and disrupt circadian rhythms [14]. In this context, these misalignments have been found to contribute to metabolic diseases such as obesity and metabolic syndrome (MetS) [15]. MetS is a heterogeneous disorder characterized by a cluster of different pathologies, such as abdominal obesity, hypertension, dyslipidemia, or insulin resistance, which can increase the risk of suffering type II diabetes, cardiovascular diseases, or nonalcoholic fatty liver disease (NAFLD), among others [16]. In addition, obesity can cause the enlargement (i.e., hypertrophy) and multiplication of adipocytes (i.e., hyperplasia), resulting in harmful changes in the adipose tissue phenotype. All of this, in turn, leads to a chronic, low-grade inflammatory state which can increase the production of reactive oxygen species (ROS), which accumulate and generate oxidative stress that damages cells and tissues [17]. In this regard, it has been recently described that there is a bidirectional relationship between oxidative stress and circadian dysregulation, wherein misaligned cues disrupting circadian rhythms can induce oxidative stress and vice versa [18,19]. 

For these reasons, mammals have developed antioxidant systems to protect themselves against the harmful effects of free radicals. Thus, the first line of defense against oxidative stress are the enzymatic antioxidant defenses. In this scenario, the NRF2-KEAP1-ARE pathway is of special relevance, since it plays a central role in inducing the antioxidant response at a genomic level. Under normal conditions, NRF2 is repressed by its natural inhibitor, KEAP1. However, NRF2 translocates to the nucleus in response to oxidative stress, where it binds to the antioxidant response element (ARE) sequences, increasing the expression of several antioxidant genes [20,21]. Among its targets, the most notable are the superoxide dismutases (SODs), including SOD1 (cytoplasmic) and SOD2 (mitochondrial), which detoxify the superoxide anion; glutathione peroxidase (GPx1) and glutathione-disulfide reductase (GSR), related to glutathione (GSH) homeostasis; and NAD(P)H quinone oxidoreductase 1 (NQO1), sestrin-2 (SESN2), and heme-oxygenase 1 (HO-1), which play a key role in the enzymatic detoxification of free radicals [22,23,24,25]. Furthermore, our bodies possess an enzyme-independent antioxidant system constituted of compounds such as GSH, vitamins C and E, and metabolites such as taurine or α-ketoglutaric acid [26,27,28]. Interestingly, biological rhythms have also been closely related to oxidative stress responses in organisms [29]. In fact, it has been described that the expression of the nuclear factor erythroid-derived 2-like 2 (*Nfe2l2*), the gene that encodes NRF2, occurs under the transcriptional regulation of the clock system proteins BMAL1 and CLOCK [30]. Additionally, diurnal oscillations of GSH levels or *Sod1* or *Ho-1* gene expressions have been described in the liver of healthy mice [31]. Consequently, misaligned cues could also have an impact on the antioxidant response. 

Natural antioxidants in food have gained attention for their ability to counteract the effects of free radicals in the body. As many of the chemical compounds serving as antioxidants cannot be synthesized by mammals, they must be obtained from the diet. In this sense, phenolic compounds, mainly found in vegetables, fruits, nuts, cereals, or beverages such as tea or red wine, have become particularly important [32,33]. In this regard, the supplementation with a grape seed proanthocyanidin extract (GSPE), primarily composed of flavonoids, such as catechin or epicatechin and obtained from grape seeds, has demonstrated their antioxidant properties by modulating the gene expression of antioxidant enzymes in vitro and increasing the activity of antioxidant enzymes such as SOD in vivo [34,35]. However, GSPE supplementation not only affects the antioxidant system but also has beneficial effects at the metabolic level. In line with this, the oral administration of GSPE to rats fed a cafeteria diet (CAF), a model of MetS, showed a reduction in body weight gain, improved lipid metabolism, and lowered blood pressure [36,37]. Interestingly, it has been described that the effects of GSPE could also be mediated by their influence on circadian machinery. In this context, our research group has been working on elucidating these mechanisms, and we have shown the modulation of *Bmal1* and *Nampt* gene expression in both central and liver peripheral clocks promoted by GSPE supplementation [38,39]. Furthermore, GSPE was reported to improve the metabolic status after light/dark cycle disruption [40]. More recently, we have also shown that GSPE administration restored the diurnal rhythms of antioxidant enzyme gene expression lost by the CAF in the liver [19]. 

Taking into account the above mentioned, this study aimed to investigate how a sudden shift in the photoperiod impacts on the modulation of the liver antioxidant metabolism in healthy and diet-induced obese rats and the effect of GSPE oral administration during this sudden change. To this end, photoperiod-sensitive strain Fischer 344/IcoCrl rats were long-term fed a standard diet (STD) or a CAF. One week before euthanasia, rats were transferred from a standard photoperiod (12 h light/12 h darkness) to a short photoperiod (6 h light/18 h darkness, L6) or to a long photoperiod (18 h light/6 h darkness, L18). During this time, they were also administered either a vehicle (VH) or GSPE diluted in VH. Body weight was measured weekly, and the moment before euthanasia, serum and liver were collected and kept at −80 °C before further processing for biochemical parameters, hepatic antioxidant-related gene transcription, protein expression, and metabolomics analysis.

## 2. Results

### 2.1. Abrupt Photoperiod Changes Altered Body Weight Gain, While Treatment with GSPE Partially Improved Serum Biochemical Parameters Affected by the CAF

**Figure 1:**
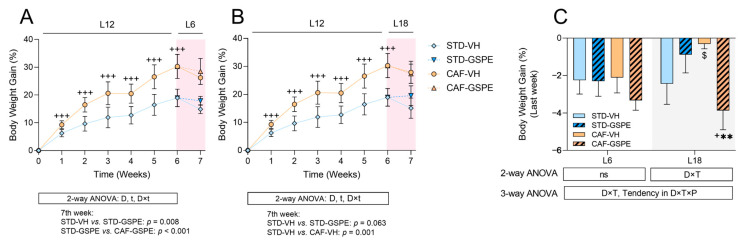
Body weight gain (% to week 0): (**A**) body weight gain in the L6 condition; (**B**) body weight gain in the L18 condition; (**C**) percentage of body weight gain during the last week and the moment before euthanasia in the experiment. Values are expressed as the mean ± S.D. (**A**,**B**) or as the mean ± S.E.M. (**C**); *n* = 5–6 for the L6 and L18 conditions. The statistical analyses for the figures in (**A**,**B**) were performed using 2- and 3-way repeated measures ANOVAs, and for the figure in (**C**) using 2- and 3-way ANOVAs. The letters t, D, T, and P refer to time, diet (STD vs. CAF), treatment (VH vs. GSPE), and photoperiod (L6 vs. L18) effect, respectively. An LSD post hoc test was used to compare between groups: $ (0.1 < *p* < 0.05), + (*p* < 0.05), and +++ (*p* < 0.001) indicate differences by diet effect; ** (*p* < 0.01) indicate differences by treatment effect. ns, indicates no significance; STD, indicates standard diet-fed rats; CAF, indicates cafeteria diet-fed rats; VH, indicates rats administered vehicle; GSPE, indicates rats were administered with grape seed proanthocyanidin extract at 25 mg/kg b.w.; L6, indicates short photoperiod with 6 h light per day; L18, indicates long photoperiod with 18 h light per day.

In order to check the obesogenic effect of the CAF, rats were weekly weighted, and their body weight gain was calculated. As expected, the CAF significantly increased the body weight gain during the first 6 weeks of the experiment when compared to rats fed an STD (Figure 1A,B). In the last week of the experiment, rats were abruptly transferred from the L12 to either the L6 or L18 photoperiods and administered VH or GSPE treatment daily. The changes in the photoperiods induced homeostatic disruption in the rats that was reflected in body weight gain during the last week (Figure 1C). Regarding the L6 photoperiod, the animals showed a similar decrease in body weight gain in all groups; however, no significant differences were found due to either the diet or GSPE treatment. Regarding the L18 photoperiod, the L18-STD-VH group was severely affected, but the GSPE treatment seemed to decrease the alteration. Interestingly, the L18-CAF-VH group was less affected by the change in photoperiod, showing differences with its control diet group (*p* = 0.085). However, the GSPE treatment in the L18-CAF-GSPE group decreased the body weight gain when compared to their control diet and treatment groups (*p* = 0.018 for GSPE-treated rats, and *p* = 0.008 for CAF-fed groups). Food intake was also measured during the last week of the experiment. As shown in Figure S1, the CAF-fed rats showed an increase in food consumption regardless of the photoperiod. Interestingly, a photoperiod effect was detected when comparing between both of the CAF-VH groups, with the L18-CAF-VH group showing a lower food intake (*p* = 0.028). However, the GSPE did not reduce food intake in either of the CAF-fed groups. 

The serum biochemical parameters were also altered, especially by the CAF, as represented in Table S1. Concerning the total cholesterol (TC), diet, treatment, and photoperiod have an effect on its levels. The CAF increased the TC levels in the L18 condition, but the GSPE was able to ameliorate this significantly, reducing them in the L18-CAF-GSPE (*p* = 0.001). The triglyceride (TAG) levels were mainly affected by the diet, as shown by the CAF-fed rats’ increased levels when compared to the STD-fed rats; likewise, the GSPE treatment was able to decrease TAG levels in the L18-CAF-GSPE group when compared to the L18-CAF-VH group (*p* = 0.004). The nonesterified fatty acids (NEFAs) levels were also highly altered by the CAF, especially in the L18 condition; however, the GSPE treatment showed no effects on this parameter. As expected, glucose levels were also affected by the diet, with increased levels in the CAF-fed rats when compared to the STD-fed rats; but the GSPE showed no effects. Finally, insulin levels were mainly affected by diet and treatment in the L6 condition. The L6-CAF-VH group showed increased levels for this parameter, but the GSPE treatment significantly decreased its levels (*p* = 0.005) in the L6-CAF-GSPE group, showing similar values to those of the STD-fed rats. 

### 2.2. CAF Highly Impacted Hepatic Biochemical Parameters, with GSPE Reducing Liver Weight and Total Lipid Content in the L6 Condition

To assess the biochemical status of the liver in the different experimental groups, we carried out the measurement of the liver weight, total lipid content, TC, TAG, and phospholipids (Table 1). Regarding the liver weight, in the L6 condition, the CAF-fed rats groups showed a significantly increased liver weight when compared to their diet controls (*p* = 0.003 between the VH groups and *p* = 0.029 between the GSPE-treated rats). A similar effect was also observed in the L18 condition, in which the CAF increased the liver weight when compared to the STD-fed groups (*p* = 0.044 between the VH groups and *p* = 0.050 between the GSPE-treated rats). This observation agreed with the effects observed in the hepatic total lipid content. Thus, the CAF highly increased the total lipid content. In more detail, in the L6 condition, the L6-CAF-VH group showed more than two-fold the lipid content when compared to the L6-STD-VH group (*p* < 0.001); interestingly, the GSPE treatment was able to decrease the lipid content despite the CAF (*p* = 0.002). In the L18 condition, the total lipid content also increased in both of the CAF-fed groups when compared to their diet controls, with those differences being statistically significant only among the GSPE-treated groups (*p* = 0.014). Interestingly, a photoperiod effect was also observed in the CAF-VH groups, being statistically lower in L18 when compared to L6 (*p* = 0.003). 

Regarding the biochemical parameters, the CAF increased the TC levels in both the L6 and L18 conditions when compared to the STD-VH groups (*p* = 0.044 for L6 and *p* = 0.012 for L18 condition), showing similar levels in both of the photoperiod conditions; however, the GSPE was unable to reduce the TC levels. Regarding the TAG levels, no differences were found due to the diet or treatment in the L6 condition. However, in the L18 condition, the L18-CAF-GSPE group showed increased TAG levels when compared to its diet control (*p* = 0.008). Interestingly, there were an increase in the TAG levels in the CAF-GSPE group in the L18 condition when compared with the same group in the L6 condition (*p* = 0.037). Regarding the phospholipid levels, they were notably altered in both of the CAF-fed groups in both photoperiod conditions in comparison with their dietary controls, showing significance in the L6 condition (*p* = 0.002 for the VH-treated rats and *p* > 0.001 for the GSPE-treated rats in the L6 condition; *p* = 0.088 for GSPE-treated groups in the L18 condition). Interestingly, the STD-GSPE group in L18 had notably increased phospholipid levels when compared to the same group in the L6 condition (*p* = 0.016 for GSPE-treated groups). Altogether, the measured parameters were notably modified by the diet in both photoperiods. However, the treatment with GSPE could alleviate the increase in the total lipid content in the L6 condition promoted by the CAF.

### 2.3. GSPE Treatment Increased Antioxidant Response Gene Expression in CAF-Fed Rats in a Photoperiod-Dependent Manner

**Figure 2:**
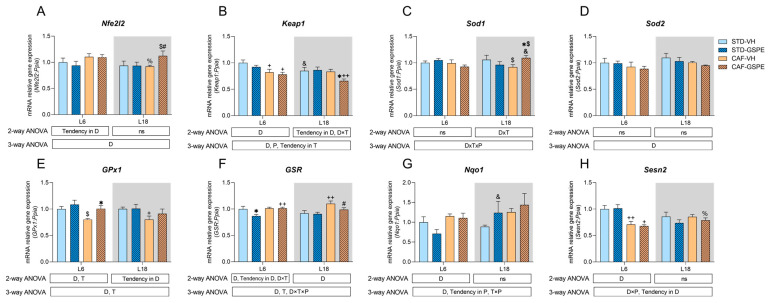
Antioxidant-related relative gene expression in the liver. All data were relativized to the L6-STD-VH group: (**A**) nuclear factor erythroid-derived 2-like 2 (*Nfe2l2*); (**B**) Kelch-like ECH-associated protein 1 (*Keap1*); (**C**) superoxide dismutase 1 (*Sod1*); (**D**) superoxide dismutase 2 (*Sod2*); (**E**) glutathione peroxidase 1 (*GPx1*); (**F**) glutathione-disulfide reductase (*GSR*); (**G**) *NAD*(*P*)*H dehydrogenase* (*quinone 1*) (*Nqo1*); (**H**) *Sestrin-2* (*Sesn2*). Values are expressed as the mean ± S.E.M.; *n* = 4–6 for the L6 and L18 conditions. The statistical analyses were performed using 2- and 3-way ANOVAs. The letters D, T, and P refer to diet (STD vs. CAF), treatment (VH vs. GSPE), and photoperiod (L6 vs. L18) effect, respectively. An LSD post hoc test was used to compare between groups: $ (0.1 < *p* < 0.05), + (*p* < 0.05), and ++ (*p* < 0.01) indicate differences by diet effect; # (0.1 < *p* < 0.05) and * (*p* < 0.05) indicate differences by treatment effect; % (0.1 < *p* < 0.05) and & (*p* < 0.05) indicate differences by photoperiod effect. ns, indicates no significance; STD, indicates standard diet-fed rats; CAF, indicates cafeteria diet-fed rats; VH, indicates rats administered vehicle; GSPE, indicates rats were administered with grape seed proanthocyanidin extract at 25 mg/kg b.w.; L6, indicates short photoperiod with 6 h light per day; L18, indicates long photoperiod with 18 h light per day.

To investigate how the abrupt change in the light/dark cycle affects the antioxidant response in the liver in a healthy and obese context, we assessed the gene expression of various antioxidant genes. First, we examined the expression of *Nfe2l2*, which encodes for NRF2, a key transcription factor involved in the antioxidant response in the organism (Figure 2A). Regarding the L18 condition, the expression in the L18-CAF-GSPE was upregulated due to the diet and treatment, as shown by the comparisons of this group with the L18-STD-GSPE group (*p* = 0.070) and with the L18-CAF-VH group (*p* = 0.063). Furthermore, we assessed the gene expression of the natural inhibitor of NRF2, *Keap1.* Its expression was affected by diet, photoperiod, and treatment (Figure 2B). Concerning the L6 condition, *Keap1* expression was downregulated by the CAF, showing differences when comparing the L6-CAF-VH with L6-STD-VH groups (*p* = 0.016) and L6-CAF-GSPE vs. L6-STD-GSPE (*p* = 0.048). In addition, only the group L18-CAF-GSPE led to a downregulation of the *Keap1* gene expression in comparison to the L18-CAF-VH group (*p* = 0.023) in this photoperiod condition. Moreover, the L18-CAF-GSPE group also showed differences with its diet control (*p* = 0.008). Additionally, we also detected a photoperiod effect in the STD-VH groups that downregulated *Keap1* expression in the L18 condition (*p* = 0.037). 

To investigate additional antioxidant pathways targeted by NRF2, we examined the gene expression of two proteins involved in the detoxification of the superoxide anion, *Sod1* and *Sod2,* mainly located in cytoplasm and in mitochondria, respectively. Regarding the *Sod1* expression, we found no relevant effects in the L6 condition (Figure 2C). Interestingly, the L18-CAF-GSPE group showed an increased *Sod1* expression when compared to the L18-STD-GSPE group (*p* = 0.087) and to the L18-CAF-VH group (*p* = 0.033), as well as when compared to the same group in the L6 condition (*p* = 0.041). Regarding the *Sod2* expression, it showed no significant differences by diet, treatment, or photoperiod, (Figure 2D). 

Additionally, we further evaluated the status of the enzymes also targeted by NRF2 and related to the glutathione (GSH) homeostasis, such as *GPx1,* which is in charge of detoxifying hydrogen peroxide using GSH, and *GSR*, which reduces the oxidized GSH. Regarding the *GPx1* expression, it showed significant differences by diet and treatment (Figure 2E). Interestingly, both of the GSPE-treated groups showed upregulated *GPx1* expression when compared to their control treatment, with the differences found between the L6-CAF-GSPE and the L6-STD-GSPE groups (*p* = 0.040) being statistically significant. Similarly, in the L18 photoperiod, there was a significant decrease in the *GPx1* gene expression in the L18-CAF-VH group compared to the L18-STD-VH group (*p* = 0.046). Concerning the *GSR* expression, it was mainly affected by diet and treatment (Figure 2F). In the L6 condition, the L6-STD-GSPE group showed a decreased expression when compared with the L6-STD-VH group (*p* = 0.008). In the L18 condition, the L18-CAF-VH group exhibited increased expression when compared to the L18-STD-VH (*p* = 0.003). However, the GSPE treatment in the CAF-fed rats seemed to downregulate its expression, as shown when comparing the L18-CAF-GSPE group to the L18-CAF-VH group (*p* = 0.054).

The expressions of *Nqo1* and *Sesn2*, additional NRF2 targets, were also assessed because of their cytoprotective role in cells against ROS. In the first case, its gene expression was mainly altered by diet (Figure 2G); however, no differences were found among the groups. Notably, we found a photoperiod effect among the STD-GSPE groups, with an increase in its expression in the L18 condition when compared to the L6 condition (*p* = 0.035). Regarding *Sesn2*, the CAF downregulated its expression in both groups in the L6 condition (*p* = 0.009 for the VH-treated groups, and *p* = 0.041 for the GSPE-treated groups), but no more significant differences were found when comparing among the groups (Figure 2H). 

### 2.4. CAF Significantly Altered Both HO-1 and SOD1 Expressions after an Abrupt Change in Photoperiod, and GSPE could Modulate HO-1 Expression in a Photoperiod-Dependent Manner

**Figure 3:**
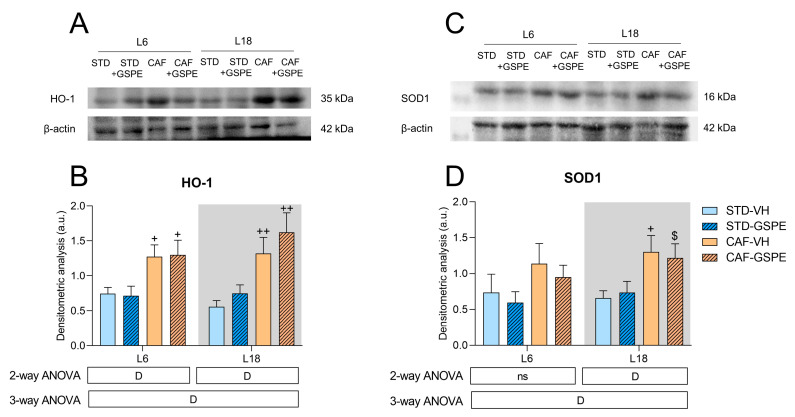
Effects of diet and the sudden light/dark cycle disruption in hepatic HO-1 and SOD1: (**A**) representative Western blot analysis of HO-1; (**B**) representative densitometric analysis of HO-1 normalized to β-actin (housekeeping protein) in liver; (**C**) representative Western blot analysis of SOD1; (**D**) representative densitometric analysis of SOD1 normalized to β-actin (housekeeping protein) in liver. Values are expressed as the mean ± S.E.M.; *n* = 4 for the L6 and L18 conditions. The statistical analyses were performed using 2- and 3-way ANOVAs. The letter D refer to diet (STD vs. CAF) effect. An LSD post hoc test was used to compare between groups: $ (0.1 < *p* < 0.05), + (*p* < 0.05), and ++ (*p* < 0.01) indicate differences by diet effect. ns, indicates no significance; STD, indicates standard diet-fed rats; CAF, indicates cafeteria diet-fed rats; VH, indicates rats administered vehicle; GSPE, indicates rats were administered with grape seed proanthocyanidin extract at 25 mg/kg b.w.; L6, indicates short photoperiod with 6 h light per day; L18, indicates long photoperiod with 18 h light per day.

To further explore the antioxidant response in the present experimental model, we evaluated the expression of SOD1 and HO-1, target genes of NRF2 that are involved in reducing oxidative stress and inflammation, using Western blotting. As shown in Figure 3A, CAF strongly altered the expression of HO-1, showing higher levels in the CAF-fed rats when compared to the STD-fed rats in both photoperiods. The densitometric analysis (Figure 3B) confirmed these alterations caused by the diet. In the L6 condition, the expression of HO-1 significantly increased because of the diet when comparing both the CAF-fed groups with their STD-fed control groups (*p* = 0.036 for the VH-treated groups, and *p* = 0.022 for the GSPE-treated rats). Similarly, in the L18 condition, both CAF-fed groups showed higher levels of HO-1 expression than the STD-fed groups (*p* = 0.004 for the VH-treated rats, and *p* = 0.004 for the GSPE-treated rats). Interestingly, the animals treated with the GSPE seemed to have increased HO-1 expression when compared to their treatment control groups in the L18 condition. 

In a similar way, SOD1 expression was also highly altered because of the CAF, as shown in Figure 3C. Indeed, the densitometric analysis (Figure 3D) confirmed this alteration. Concerning the L18 condition, both of the CAF-fed groups also showed increased levels of SOD1, especially in the L18-CAF-VH group (*p* = 0.032) and, interestingly, the GSPE treatment seemed to decrease its expression in rats fed the CAF in the L6 photoperiod.

### 2.5. GSPE Improved Liver Antioxidant Metabolic Profile of CAF-Fed Rats after an Abrupt Change in Photoperiod

**Figure 4:**
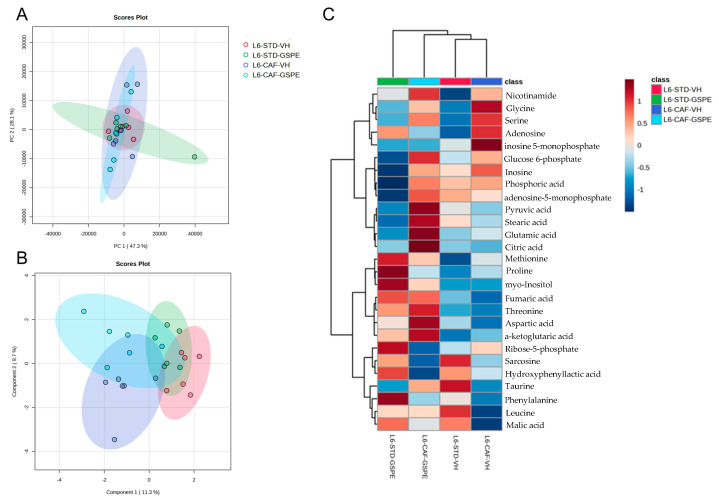
Metabolomic analysis of liver antioxidant-related metabolites in the L6 condition: (**A**) principal component analysis (PCA), (**B**) sparse partial least squares discriminant analysis (sPLS-DA), and (**C**) heatmap analysis of antioxidant-related detected metabolites in the liver metabolomics of rats transferred to the L6 photoperiod. STD, rats fed a standard diet; CAF, rats fed a cafeteria diet; VH, rats administered vehicle; GSPE, indicates rats were administered with grape seed proanthocyanidin extract at 25 mg/kg b.w.; L6, indicates short photoperiod with 6 h light per day.

**Figure 5:**
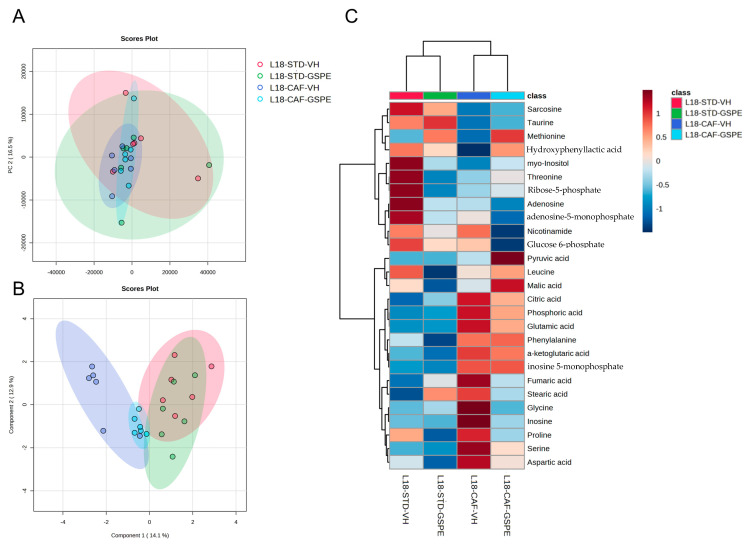
Metabolomic analysis of liver antioxidant-related metabolites in the L18 condition: (**A**) principal component analysis (PCA), (**B**) sparse partial least squares discriminant analysis (sPLS-DA), and (**C**) heatmap analysis of antioxidant-related detected metabolites in the liver metabolomics of rats transferred to the L18 photoperiod. STD, rats fed a standard diet; CAF, rats fed a cafeteria diet; VH, rats administered vehicle; GSPE, indicates rats were administered with grape seed proanthocyanidin extract at 25 mg/kg b.w.; L18, indicates long photoperiod with 18 h light per day.

To better understand the antioxidant response of the animals, we analyzed 27 metabolites related to liver oxidative stress. Figure 4 and Figure 5 represent the metabolomic profile in both the L6 and L18 conditions, using principal component analysis (PCA), sparse partial least squares discriminant analysis sparse least (sPLS-DA), and heatmap analysis, while the antioxidant metabolites annotated are shown in Table S1. Regarding the L6 condition, the PCA did not show any clustering among groups (Figure 4A). However, the sPLS-DA showed clustering by diet with a clear separation between the STD and the CAF-fed groups (Figure 4B). The antioxidant-related metabolomic profile (Figure 4C) showed differences depending on both factors: diet and treatment. Regarding the differences between diets, the STD-fed rats showed increased levels of metabolites, such as malic acid, sarcosine, or hydroxyphenyl lactic acid, while the CAF-fed groups showed increased levels of nicotinamide, glycine, or serine, with both glycine and serine being involved in glutathione (GSH) synthesis. Regarding the differences between treatment, it seemed that the GSPE treatment increased the hepatic antioxidant-related metabolites in both groups but especially in the L6-CAF-GSPE group. In this case, the GSPE-treated rats showed increased metabolite concentration of fumaric acid or threonine, while the VH-treated rats showed reduced levels of these metabolites (Table S2). Additionally, these differences were higher when comparing between the CAF-fed rats, with increased levels of almost all metabolites analyzed in the GSPE-treated group. 

On the other hand, in the L18 condition, the PCA did not show any clustering among groups (Figure 5A). In addition, as before, the sPLS-DA did show a marked clustering by diet (Figure 5B). Interestingly, the L18-CAF-GSPE group seemed to get closer to the STD-fed groups while moving farther away from the L18-CAF-VH group. The heatmap analysis (Figure 5C) shows the antioxidant-related metabolomic profile, which also clustered by diet. In the STD-fed rats, higher levels of taurine and sarcosine were observed, whereas citric acid, inosine-5-monophosphate, or glutamic acid, also involved in GSH synthesis, were the main metabolites in the CAF-fed rats (Table S2). In this case, the treatment with GSPE did not increase the concentration of metabolites when compared to the VH-treated groups.

## 3. Discussion

Biological rhythms play a vital role in regulating physiological, behavioral, and metabolic processes to help organisms adapt to their environment through external cues or ZT. Thus, a precise regulation is necessary, since its disruption has been linked to the debut and/or progression of multiple disorders, many of which are related to metabolism and oxidative stress (e.g., overweight, type 2 diabetes, or MetS) [41]. Obesity, a key comorbidity of MetS, is closely related to the production of ROS in the organism. Additionally, ROS is also used as a signal transductor for storing triglycerides in adipose tissue [42]. However, in an obesity context, such as that induced by the CAF, adipose tissue is also responsible for releasing adipokines that increase the production of free radicals. Consequently, the alteration in the metabolism of oxygen, added to the reduction in the activity of antioxidant enzymes, can lead to several abnormalities, promoting an imbalance between oxidant and antioxidant molecules [43]. In this regard, certain natural bioactive compounds, including GSPE, have demonstrated their efficacy in restoring circadian machinery and alleviating symptoms and comorbidities associated with metabolic disorders such as obesity or MetS. Additionally, GSPE exhibits antioxidant properties by scavenging ROS and increasing the activity of antioxidant enzymes within the body. Therefore, we aimed to describe the impact of misaligned cues, such as the sudden photoperiod shift or the influence of the CAF, and to report the potential ability of GSPE in the attenuation of these effects on the hepatic oxidative-related mechanisms. In this sense, as it has been previously shown, the disturbance in the light/dark cycle, along with the CAF, resulted in multiple dysregulations in the animals. Interestingly, the oral administration of the GSPE partially restored these misalignments in a photoperiod-dependent manner, suggesting a potential chronotherapeutic effect of the GSPE.

The CAF has been widely used to induce obesity and MetS by providing human-like unhealthy food high in energy and palatability, such as bacon, biscuits with cheese or pâté, or even pastries, replicating human eating patterns [44]. As expected, Figure 1 shows that rats fed a CAF exhibited significantly increased body weight gain compared to rats fed an STD during the first six weeks of the present experiment. Moreover, it has also been described that CAF increases appetite, as it contains a high quantity of sugars and salt, thereby promoting hyperphagia [45]. This fact was also observed in our study, as CAF-fed rats presented hyperphagia compared to STD-fed rats, which was evidenced in our model, since the CAF-fed rats significantly showed increased food intake compared to the rats fed an STD. 

In the last week of our experimental model, the rats were abruptly transferred from the L12 photoperiod to the L6 or L18 photoperiod, in which animals received a daily oral dose of either VH or GSPE. This chronodisruption metabolically altered the rats in a photoperiod-dependent manner, since globally the rats transferred to L6 showed a decrease in body weight of around 2%; nevertheless, this pattern was not followed by rats under the L18 condition. The CAF also promoted dyslipidemia, a MetS comorbidity, especially in the L18 condition, since these rats showed increased serum levels of cholesterol, triglycerides, and NEFA, as well as glucose and insulin. In contrast, rats in the L6 condition exhibited elevated levels of triglycerides, glucose, and insulin, as shown in Table S1. These results were also aligned with the results previously described by other authors [36,45,46]. Furthermore, the CAF effects appeared to be exacerbated by the alteration of the light/dark cycle, since it usually appears as an increase in body weight gain, as well as dysregulation of the serum biochemical parameters, as described by many authors [10,47]. However, in agreement with our present results, other chronobiological studies have reported no significant differences in body weight gain between groups or a reduced body weight gain after the misaligning cue when compared to the control groups [9,48]. 

In our study, rats were euthanized one week after disrupting their light/dark cycle to avoid adaptations to the new photoperiods, as the re-entrainment of the internal clocks can take up to 10 days. This could explain the interesting responses found in each photoperiod condition (L6 and L18) and might represent an important factor that could have an influence on the present experimental results [49]. Additionally, previous studies have reported differences in body weight gain in the Fischer 344 rats based on the used photoperiod, in which rats under photoperiods of more than 12 h of light per day presented an increased body weight gain, as well as photoperiod-dependent changes in serum biochemical parameters. These results are in agreement with our present findings [50,51,52]. 

During the last week, rats received a daily oral dose of either VH or GSPE. No effects were observed in body weight gain concerning the L6 condition, while the GSPE treatment reduced serum insulin levels in the CAF-fed rats. On the other hand, the GSPE treatment in the L18 condition showed differential effects based on the diet, resulting in decreased body weight gain in the rats fed a CAF, which was accompanied by decreased levels of serum cholesterol and triglycerides (Table S1). Thus, it is likely to suggest that in our study the GSPE treatment exhibited a photoperiod-dependent effect. This could be explained because the effects of the GSPE are highly dependent on its bioavailability, which, in turn, is highly influenced by the composition of the gut microbiota. Consequently, our discoveries can be, at least, partially attributed to the impact of both diet and photoperiod on the gut microbiota [53]. In this regard, it has been previously shown by our research group that gut microbiota is significantly altered in groups fed a CAF and exposed to an L18 photoperiod. This fact is in agreement with the L18-CAF-GSPE group of the present study, which exhibited the greatest reduction in body weight gain. Moreover, these effects could also be mediated by the (-)-epigallocatechin-3-gallate (EGCG), found in GSPE, as it has been previously described to decrease body weight and TAG levels in HFD-fed mice [54].

Additionally, some altered results were also observed in the studied liver-related parameters, as shown in Table 1. In this sense, regardless of the photoperiod, the CAF notably altered the liver weight, as well as all the hepatic biochemical parameters measured, including the total lipid content, TC, TAG, and phospholipids. These results are in accordance with other studies that exhibited an increase in hepatic lipid-related parameters promoted by the CAF [55,56,57]. Therefore, the maintenance of lipid accumulation in the liver promoted by the CAF could, ultimately, lead to NAFLD, since hepatic lipid accumulation is a comorbidity of this pathology [58]. Furthermore, it is noteworthy to mention that the GSPE treatment was able to significantly decrease the total lipid content in the liver, which was accompanied by a slight reduction in the liver weight in the L6 condition. Additionally, the disruption of the circadian rhythm resulted in an increase in the phospholipid content in the STD-fed rats during L18 in comparison to L6. In this sense, the liver plays a key role in lipid metabolism, as it synthetizes fatty acids, which are subsequently converted to TAG, promoting de novo lipogenesis [59]. It has been described that the lipid accumulation on hepatocytes of patients suffering NAFLD is mainly occasioned by a dysregulation in this process [60]. The main protein in charge of regulating de novo lipogenesis is SREBP1c, which induces the expression of lipogenic genes. However, its regulation should be precise, as its overexpression in liver has shown a two-fold increase in hepatic TAG [61]. Furthermore, circadian machinery regulates this protein, as diurnal rhythmicity in its expression has been reported [60]. Therefore, it can be hypothesized that in the presence of the CAF, the circadian disruption promoted by altering the light/dark cycle could perturbate the diurnal rhythmicity of SREBP1c, leading to its possible activation, which could increase the hepatic lipid content, and, additionally, that this phenomenon could be different depending on the photoperiod. Furthermore, our results are consistent with the findings obtained by Rodríguez and colleagues, who demonstrated that the hepatic lipid metabolism was influenced by the photoperiod in rats fed an STD, resulting in modified lipid content determined by the length of the photoperiod [62]. Additionally, this study also showed a GSPE influence on these parameters, which was also conditioned by the photoperiod. Thus, the disturbance in the light/dark cycle, combined with the effect of the photoperiod on the hepatic lipid metabolism, might explain some of the changes observed in the hepatic-lipid-related parameters examined in our investigation.

Another harmful effect of high-fat diets such as the CAF is their critical role in promoting the dysregulation of the circadian machinery, which altogether can lead to a condition of oxidative stress [12,18,63]. In addition, circadian machinery is also involved in regulating the antioxidant response within the organism, as the expression and/or the activity of many antioxidant enzymes fluctuate in a circadian manner or are regulated by the circadian clock genes, with NRF2 playing a pivotal role in linking both oxidative stress and circadian rhythms [29,30,64,65]. Our findings, as exhibited in Figure 2, show a slight increase in the expression of *Nfe2l2* accompanied by a significant decrease in the expression of its inhibitor, *Keap1*, in the CAF-fed rats in the L18 condition. Contrarily, CAF-fed rats in the L6 condition showed no differences with the STD-fed rats in the expression of both genes. In this context, NRF2 translocates into the nucleus and increases the expression of several antioxidant-related genes under an oxidative stress condition [66]. Therefore, the results obtained could reflect an attempt to maintain the redox homeostasis within the organism after an oxidative stress condition promoted by both the CAF and the light/dark cycle disruption. The treatment with the GSPE resulted in an increase in the NRF2-mediated antioxidant response in the L18 condition, as it tended to upregulate the expression of *Nfe2l2*, accompanied by a significant downregulation in the expression of *Keap1*, suggesting increased levels of NRF2. These results are in concordance with the significant upregulation found in the *Sod1* expression, as the expression of this gene is modulated by NRF2. In agreement with our results, several studies, with diverse experimental designs, including mice, rats, and pigs, have shown the activation of NRF2 promoted by GSPE administration [67,68,69]. In this context, the protocatechuic acid, found in GSPE, may be responsible for the upregulation of NRF2, as its ability to activate this protein has been reported [70,71]. Furthermore, it has been shown that GSPE also has influence in the circadian machinery, acting as a modulator of some clock gene expressions, including *Bmal1* [38,72]. In fact, our group has recently described that the oral administration of GSPE was able to restore the diurnal rhythm of *Bmal1* lost by the CAF in a study of circadian rhythm under an obesogenic context [73]. Moreover, the expression of NRF2 is under the control of the circadian system, as it is regulated by BMAL1 [65]. Thereby, GSPE could act by restoring the *Bmal1* diurnal rhythm, which could have an effect in *Nfe2l2* expression and, subsequently, an increase in the activity of the NRF2/KEAP1/ARE pathway that upregulates the expression of *Sod1* and *GPx1*. Interestingly, the increase in NRF2 promoted by the GSPE treatment in L18 was accompanied by a decrease in serum TC and TAG. In this regard, there is increasing evidence that NRF2 plays a key role in hepatic fatty acid metabolism, as it has been speculated that NRF2 inhibits lipogenesis and enhances fatty acid oxidation to protect the liver from steatosis [59]. Therefore, the improvement in the serum lipid profile detected by the decreased levels of TC and TAG could be also mediated by the increase in NRF2 promoted by the GSPE. 

On the other hand, the GSPE treatment enhanced the antioxidant response by increasing *GPx1* expression in the L6 condition. GPx1 plays an important role in the antioxidant response, since it is responsible for the reduction in hydrogen peroxide via GSH [74]. As part of the enzymatic antioxidant-response mechanism, GPx1 is also under the control of the circadian machinery. In fact, its expression has been described to follow a diurnal rhythmicity [29]. In line with this, our group recently described the restoration of the diurnal rhythmicity of *GPx1,* lost by the CAF, through GSPE treatment in a time-of-day dependent manner. This mechanism could explain the increase in the expression of this gene and the enhancement of the antioxidant response [19]. However, the expressions of two main targets of NRF2, including *Sod2*, the mitochondrial isoform of SOD, and *Sesn2*, which is fundamental against oxidative stress, remained unresponsive to the GSPE treatment, as no differences were found regardless of the photoperiod condition. 

**Figure 6:**
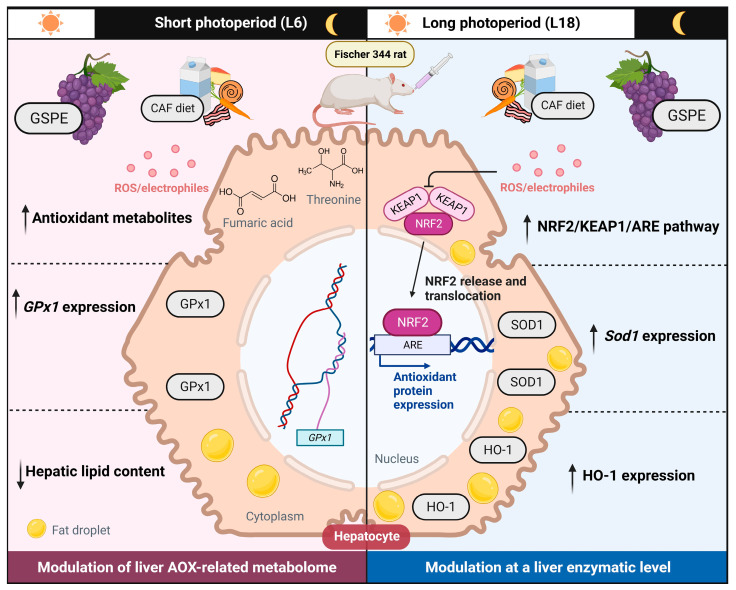
Potential antioxidant mechanisms of action of GSPE to alleviate the effects of a sudden change in the photoperiod on the liver of diet-induced obese rats. In the L6 condition, the GSPE treatment in the CAF-fed rats increased antioxidant metabolites and the expression of *GPx1*, and it reduced the hepatic lipid content. On the other hand, the treatment with GSPE in the L18 condition promoted the activation of the NRF2/KEAP1/ARE pathway, which involved the transcription of several antioxidant genes, including Sod1. Moreover, this route also increased the expression of the inducible heme-oxygenase, HO-1. Up arrow: increased; down arrows: reduced. Created with BioRender.com (accessed on 24 November 2023).

The expression of HO-1 and SOD1 is determined by the activation of the NRF2 pathway, which is primarily activated in an oxidative context. Both proteins showed differences specially in terms of the influence of the CAF on the metabolism, as shown in Figure 3. In this sense, the CAF increased the expression of HO-1 regardless of the photoperiod. Heme-oxygenases catalyze the breakdown of heme, generating carbon monoxide, biliverdin, and iron. Heme is present in many crucial proteins, including hemoglobin and cytochromes. Nevertheless, free heme is highly cytotoxic due to its ability to increase ROS production. Consequently, it needs to be quickly metabolized to prevent cellular damage [75]. In this sense, HO-1 is the inducible isoform of heme-oxygenases, with its expression being upregulated in an oxidative stress condition due to its antioxidant properties [76]. Therefore, the increase in HO-1 expression observed in the CAF-fed rats could be a response to the increase in ROS production caused by the CAF in an attempt to provide protection against oxidative stress and to maintain the redox homeostasis. Interestingly, the GSPE administration in the CAF-fed rats in the L18 condition showed a notable increase in HO-1 expression when compared to its treatment control group. This fact could be explained as NRF2 is the major transcriptional activator of HO-1 [76]. Consequently, the increase in NRF2 promoted by the GSPE administration in the L18-CAF-GSPE group can be the basis of the increase in the HO-1 protein expression observed. On the contrary, the GSPE treatment was not able to increase HO-1 in the L6 condition, as it could not increase the expression of *Nfe2l2*. Therefore, it could be suggested that the GSPE showed a photoperiod effect by hypothetically activating the NRF2/KEAP1/ARE signaling pathway, mainly in the L18 condition, which could be related to the increase in gene expression previously described and HO-1 protein expression, as explained in Figure 6.

On the other hand, SOD1 expression was also affected by the CAF, especially in the L18 condition, where its expression was significantly upregulated by the diet. This enzyme, which is mainly located in the cytoplasm, is responsible for catalyzing the conversion of the highly reactive superoxide anion to hydrogen peroxide, a less reactive free radical [27]. Therefore, the increase in the expression of this protein in the CAF-fed groups could be attributed to the increase in free radicals promoted by the CAF. However, the GSPE treatment was not able to increase the expression of this protein regardless of the photoperiod. In this sense, the differences shown in the SOD1 gene and protein expression could be hypothetically explained due to the post-translational regulation of SOD1. Indeed, it has been reported the existence of two species of SOD1 mRNA in vitro whose only difference is in the length of the 3′UTR region, where regulatory elements are located, and these transcripts produce different quantities of SOD1 [77]. Additionally, it has also been demonstrated that miR-206 can target the 3′-UTR of SOD1 diminishing in consequence the half-life of the SOD1 transcript [78]. Furthermore, our research group has reported that GSPE is able to importantly modulate miRNAs [79,80,81]. This could suggest a hypothetical differential effect on miRNAs’ regulation of GSPE depending on the photoperiod. In this regard, we have demonstrated that GSPE is able to modulate the hepatic molecular clock via miRNAs [82]. 

At this point, it is worth mentioning that the overexpression of HO-1 and SOD1 proteins is positively correlated with the increase in hepatic liver triglycerides due to CAF. In line with this, it has been shown that palmitate-induced lipotoxicity in vitro has been shown to occur through the generation of ROS [83,84]. 

Furthermore, hepatic antioxidant-related metabolomics were evaluated, and the metabolomic profiles showed different groups depending on the diet and photoperiod. The STD-fed rats in the L6 condition showed higher levels of malic acid, sarcosine, or hydroxyphenyl lactic acid, while the CAF-fed rats showed increased levels of nicotinamide and serine; remarkably, GSPE administration leads to an increase in liver antioxidant metabolites, such as threonine and fumaric acid, while the VH-treated rats showed decreased levels of these metabolites. Threonine is an essential amino acid that has been associated with the prevention of fat deposition, as reported after its supplementation to mice fed a high-fat diet [85,86]. In fact, this increase in threonine could also be related to the decrease in total lipids observed in the liver of these rats. However, the mechanisms involved in threonine regulation and its relationship with GSPE remain unknown. Regarding the L18 condition, the results were dependent on diet, since taurine and sarcosine were increased in the STD-fed rats while citric acid or glutamic acid were increased in the CAF-fed rats. In this sense, it seems that the liver responds to the oxidative conditions promoted by the disturbance of the light/dark cycle regardless of the diet but in a different way. However, the GSPE treatment did not show differences with the VH-treated rats. 

Concerning the present study, a noteworthy limitation may be attributed to the reduced number of animals used for each group (*n* = 6). As a result, some effects promoted by the GSPE may have been reduced or masked because of the statistical power. Regarding the effects of the GSPE, the extract underwent chemical characterization but contained a combination of several flavonoids, principally proanthocyanidins. Therefore, the study is limited by the absence of the identification of the specific pure compound or compounds responsible for the observed beneficial effects of the GSPE. Thus, for future research, it could be interesting to use pure compounds (e.g., protocatechuic acid, catechin, or gallic acid) in order to elucidate the effects of each component of the GSPE and to ensure the bioactivity of the extract in different production batches. Moreover, another limitation of the present study could be that the GSPE was used in a palliative way, when the chronodisruption promoted by the CAF and the abrupt change in the light/dark cycle was already made. Therefore, for further investigations, the GSPE could be administered in a preventive way, before the establishment of the chronodisruptors. Finally, a potential limitation of the study is the translation to human studies, as rodents have a similar but not identical metabolism to humans. Therefore, some results obtained in animals may not be replicated in human clinical trials.

## 4. Materials and Methods

### 4.1. Grape Seed Proanthocyanidins Extract (GSPE) 

The extract used in this experiment was obtained from seeds of white grapes and supplied by Les Dérives Résiniques et Terpéniques (Dax, France). The phenolic composition of the extract used was previously described by our group [87], which included catechin, epicatechin, dimers, trimers, and tetramers of procyanidins, and epicatechin gallate, among others.

### 4.2. Experimental Procedure in Rats 

**Figure 7:**
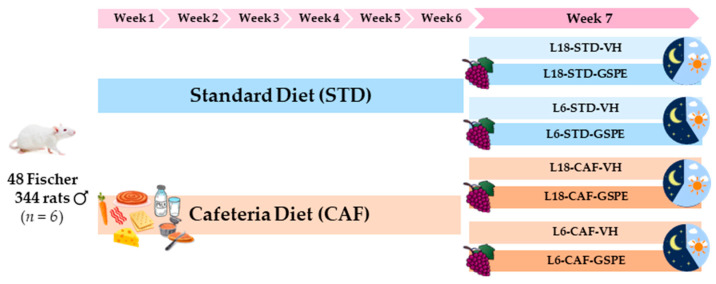
Experimental design. Forty-eight male Fischer 344 rats were randomly divided into two groups based on their diet: 24 were fed an STD and the other half were fed a CAF for 6 weeks. Then, the animals were abruptly transferred from L12 to L6 or L18 photoperiods, and treatment with VH or GSPE diluted in VH at a dose of 25 mg/kg b.w. was administered. After a week, the rats were euthanized, and serum and liver were collected for further analysis.

Forty-eight 13-week-old male Fischer 344/IcoCrl rats (Charles River Laboratories, Barcelona, Spain) were housed in pairs under standard conditions (23 °C, 55% humidity, and photoperiod, 12 h light/12 h dark; light density: 350 lux) with ad libitum access to food and drink water. We have selected F344/IcoCrl rats because they are sensitive to photoperiod cues [62,88,89,90]. After the acclimatization period, the animals underwent weighing and were subsequently assigned randomly to two groups based on their diet. Half of the animals were given an STD (kcal/100 g: 72% carbohydrates, 8% fat, and 20% protein; Safe-A04c, Scientific Animal Food and Engineering, Barcelona, Spain), while the remaining half were given a CAF (Kcal/100 g: 58% carbohydrates, 31% fat, and 11% protein) during a 6-week pretreatment period (*n* = 24). The CAF consisted of food commonly consumed by humans with high palatability and high caloric content but poor nutritional value, including biscuits with cheese and pâté, bacon, *ensaimada* (pastry), standard chow, carrot, and milk containing 22% sucrose (*w*/*v*). In the last week, the rats were transferred from the standard photoperiod (L12) to the short (L6) or long (L18) photoperiod, and treatment was orally administered, consisting of vehicle (VH, milk containing 22% sucrose, *w*/*v*) or GSPE diluted in VH (25 mg/kg b.w.), resulting in 8 groups (*n* = 6 animals per group). Throughout the course of the experiment, the weights and food intake of the rats were monitored on a weekly basis. Before euthanizing, the rats underwent a 3-h fasting period, and then they were euthanized with 3% isoflurane and decapitation. Figure 7 provides a detailed overview of the study’s design. Whole blood was collected for serum extraction following centrifugation (2000× *g* and 4 °C, 15 min). Both serum and liver samples were collected and preserved at −80 °C for subsequent analysis. All animal care and experimental procedures were approved by the Ethics Review Committee for Animal Experimentation of the Universitat Rovira I Virgili (reference number 9495). The procedures adhered to the guidelines outlined in Directive 86/609EEC of the Council of the European Union and were conducted in accordance with the protocols established by the Departament d’Agricultura, Ramaderia i Pesca of the Generalitat de Catalunya.

### 4.3. Dosage Information/Dosage Regimen

The rats were administered either a dose of VH (condensed milk diluted in water at a ratio of 1:5, *v*/*v*) or 25 mg GSPE/kg b.w. diluted in VH after the sudden photoperiod change. This particular dose of GSPE, regularly employed by our group, has been demonstrated to be the lowest but the most efficient to induce changes in key metabolic pathways in healthy rats [91]. Moreover, this dose corresponds to an intake of approximately 370 mg of phenols per day when considering the conversion from animal (rat) to human [92]. This quantity of phenols is easily attainable through (poly)phenol-rich diets such as the Mediterranean [91,92]. Both VH and GSPE were orally administered at 8 a.m. The euthanize of the rats was carried out 3 h after the last dose.

### 4.4. Hepatic RNA Extraction

For hepatic RNA extraction, 1 mL of Trizol^®^ reagent (Thermo Fisher, Madrid, Spain) was used to homogenize 20–30 mg of liver tissue in a Tissue Lyser LT (Qiagen, Madrid, Spain). Homogenate was centrifuged (12,000× *g*, 4 °C, 10 min) and supernatant was mixed with 250 µL of chloroform. Following centrifugation (12,000× *g*, 4 °C, 15 min), the aqueous phase was transferred to a new microtube. Then, 500 µL of isopropanol was added, and a centrifugation step was performed (12,000× *g*, 4 °C, 10 min). The resulting pellet was washed with 70% ethanol and centrifuged (8000× *g*, 4 °C, 5 min), repeating both steps twice. Then, 60 µL of nuclease-free water was used to resuspend the dried washed pellet and a NanoDrop 1000 spectrophotometer (Thermo Scientific, Wilmington, DE, USA) was used to assess the RNA yield and purity. 

### 4.5. cDNA Synthesis and Gene-Expression Analysis

RNA was used to synthetize cDNA using the High-Capacity cDNA Reverse Transcription Kit (Applied Biosystems, Barcelona, Spain). The reaction was performed in a Galaxy XP ClearLine Thermal Cycler (ClearLine, Dominique Dutscher, Brumath, France) according to the instructions from the manufacturer. The cDNA obtained was used to conduct a quantitative polymerase chain reaction amplification using the iTaq Universal SYBR Green Supermix (Bio-Rad, Madrid, Spain) in a CFX96 Touch Real-Time PCR Detection System (Bio-Rad, Madrid, Spain). The sequences of the oligonucleotides used in the reaction are listed in Table 2 and were synthetized by Biomers.net (Ulm, Germany). The relative expression of mRNA was depicted as a fold change and determined as a percentage of the L6-STD-VH group. The calculation was performed using the 2-∆∆Ct method, with the peptidylprolyl isomerase A (*Ppia*) gene serving as the housekeeping control, in accordance with the approach outlined by Schmittgen and Livak [93].

### 4.6. Serum Biochemical Analysis

The serum biochemical parameters glucose, nonesterified free fatty acids (NEFAs), total cholesterol (TC), and triglycerides (TAGs) were analyzed using enzymatic colorimetric assays according to the manufacturers’ instructions. The specific assays for glucose, TC, and TAG were obtained from QCA (Amposta, Tarragona, Spain), while the assay for the NEFAs was obtained from WAKO (Neuss, Germany).

### 4.7. Protein Extraction and Western Blot Analysis

RIPA buffer (50 mM Tris-HCl, 150 mM NaCl; pH 7.4, 1% Tween 20, 0.25% Na-deoxycholate) supplemented with phenylmethylsulfonyl fluoride (PMSF), protease inhibitor cocktail (PIC), and phosphatase cocktails 2 and 3 were used to homogenate the liver tissue in a Tissue Lyser LT (Qiagen, Madrid, Spain). The samples were then subjected to a 30 min shaking step, and the lysates were centrifuged (16,300× *g*, 4 °C, 15 min). The protein concentration was determined from the supernatant using the Pierce bicinchoninic acid (BCA) protein assay kit (Thermo Fisher Scientific, Madrid, Spain). A total quantity of 50 μg protein per sample underwent electrophoretic separation on 10% SDS-polyacrylamide gels (TGX FastCast Acrylamide Kit, Bio-Rad, Madrid, Spain). After electrophoretic separation, proteins were transferred to PVDF membranes in the Trans-Blot Turbo system (Bio-Rad, Madrid, Spain). The Pounceau-S staining was used to assess the efficacy of the protein transference. Membranes were then blocked with 5% nonfat milk diluted in 0.2% TBS-Tween for one hour at room temperature. Then, the following polyclonal primary antibodies in a 1:1000 dilution were used to blot the membranes overnight a 4 °C: rabbit-anti-superoxide dismutase 1 (SOD1), rabbit-anti-heme-oxygenase 1 (HO-1), and mouse-β-actin. After washing the membranes three times with 0.2% TBS-Tween, the secondary antibodies conjugated with horseradish peroxidase goat anti-mouse IgG and donkey anti-rabbit IgG (Amersham, Cytiva, Barcelona, Spain) in a 1:2000 dilution were employed to hybridized the membranes for one hour at room temperature. After three more washes, immunoreactive proteins were detected using a chemiluminescence substrate kit (Amersham ECL Select, Cytiva, Barcelona, Spain) according to the manufacturer’s instructions. Digital images were captured using a G:BOX Chemi XL1.4 (Syngene, Cambridge, UK), while the densitometry analysis was performed using ImageJ Software 1.54g (NIH, Bethesda, MD, USA). 

### 4.8. Extraction and Measurement of the Concentration of Lipids in the Liver 

To extract lipids from the liver, the Bligh and Dyer method was carried out [94]. In these lipid extracts, cholesterol, triglycerides (QCA, Amposta, Tarragona, Spain), and phospholipids (Spinreact, St. Esteve de Bas, Girona, Spain) were measured using enzymatic colorimetric assays. 

### 4.9. Metabolomics Analysis 

Rat liver samples were subjected to metabolomic analysis at the Centre for Omics Sciences (COS, Tarragona, Spain) using gas chromatography coupled with quadrupole time-of-flight mass spectrometry (GC-qTOF model 7200, Agilent, Santa Clara, CA, USA). 10–20 mg liver tissue was used for the sample extraction by adding methanol:water in a 8:2 proportion containing an internal standard mixture. Later, samples were homogenized in a bullet blender with a stainless-steel ball and incubated for 10 min at 4 °C. Following centrifugation at 19,000× *g*, supernatants underwent compound derivatization through methoximation and silylation and then evaporated to dryness. To analyze the derivatized compounds, GC-qTOF was employed. The chromatographic separation procedure was carried out according to the Fiehn method [95]. For this purpose, a HP5-MS film capillary column (30 m × 0.25 mm × 0.25 µm) was employed (J&W Scientific, Agilent, Santa Clara, CA, USA) using helium as the carrier gas in an oven program ranging from 60 to 325 °C. The ionization process was carried out by electronic impact (EI) in the full-scan mode, with 70 eV being the electron energy employed. The metabolite identification consisted in matching the EI mass spectrum and retention time to a metabolomic Fiehn library, which contained almost 1400 metabolites (Agilent, Santa Clara, CA, USA). Following putative identification of metabolites, they were semi-quantified based on an internal standard response ratio.

### 4.10. Statistical Analysis

The data expressed in both the tables and graphics are represented as the mean ± standard deviation or median and interquartile range. The normal distribution of data was probed using the Shapiro–Wilk test. The statistical tests used for the individual analyses are expressed in the figure legends. The graphics were made using Prism v. 8 software (GraphPad Software, San Diego, CA, USA), and the statistical analyses were performed using SPSS version 26 (IBM Inc., Armonk, NY, USA). A probability value of *p* < 0.005 was considered statistically significant. The metabolomic analyses, which included principal component analysis (PCA), sparse partial least squares discriminant analysis (sPLS-DA), and heatmaps, were assessed using MetaboAnalyst v.5.0 (McGill University, Montreal, QC, Canada) [96].

## 5. Conclusions

The results obtained in our study suggest that the abrupt photoperiod changes (L6 vs. L18) are differentially modulating the beneficial effects of GSPE on the hepatic antioxidant response, particularly in diet-induced obese rats. More specifically, the effect of GSPE on the L6 condition could be less mediated by an antioxidant enzymatic response (only *GPx1*) and might be more induced by modulation of the liver antioxidant-related metabolome. However, in the L18 condition, the effects of GSPE could be associated with the ability of GSPE to reduce free radicals by increasing, at least in part, the NRF2/KEAP1/ARE pathway but not by increasing the antioxidant-related metabolites in the liver. Nevertheless, further studies are needed to elucidate the underlying mechanisms by which GSPE may alleviate circadian disturbances resulting from a sudden change in the light/dark cycle at both the genomic (e.g., miRNAs) and metabolic levels.

## Figures and Tables

**Table 1:** Liver biochemical parameters.

Parameter	L6				L6 ANOVA	L18				L18 ANOVA	3-Way ANOVA
	STD-VH	STD-GSPE	CAF-VH	CAF-GSPE		STD-VH	STD-GSPE	CAF-VH	CAF-GSPE		
Liver weight (g)	12.36 ± 0.61	12.49 ± 0.59	15.29 ± 0.35 ^++^	14.5 ± 0.54 ^+^	D	13.08 ± 0.59	13.22 ± 0.95	14.92 ± 0.68 ^+^	15.1 ± 0.68 ^$^	D	D
Total lipid content (mg lipids/g liver)	33.71 ± 1.47	29.07 ± 4.28	74.15 ± 13.15 ^+++^	42.15 ± 5.65 **	D, T, Tendency in D×T	32.87 ± 3.23	31.87 ± 4.9	44.41 ± 5.23 ^&&^	55.83 ± 7.57 ^+^	D	D, T×P, D×T×P
Cholesterol (µM)	22.00 ± 3.00	23.00 ± 2.00	31.00 ± 1.00 ^+^	33.00 ± 3.00 ^+^	D	24.00 ± 2.00	24.00 ± 2.00	34.00 ± 4.00 ^+^	32.00 ± 4.00 ^$^	D	D
Triglycerides (µM)	70.00 ± 9.00	57.00 ± 8.00	65.00 ± 5.00	62.00 ± 6.00	ns	61.00 ± 6.00	55.00 ± 7.00	82.00 ± 11.00 ^$^	86.00 ± 8.00 ^++&^	D	D, D×P
Phospholipids (µM)	29.00 ± 1.00	25.00 ± 3.00	50.00 ± 2.00 ^++^	50.00 ± 5.00 ^+++^	D	41.00 ± 7.00 ^%^	41.00 ± 6.00 ^&^	52.00 ± 5.00 ^$^	52.00 ± 2.00 ^$^	Tendencyin D	D, P, Tendency in D×P

Values are expressed as the mean ± S.E.M.; *n* = 5–6 for the L6 and L18 conditions. The statistical analyses were performed using 2- and 3-way ANOVAs. The letters D, T, and P refer to diet (STD vs. CAF), treatment (VH vs. GSPE), and photoperiod (L6 vs. L18) effect, respectively. An LSD post hoc test was used to compare between groups: $ (0.1 < *p* < 0.05), + (*p* < 0.05), ++ (*p* < 0.01), and +++ (*p* < 0.001) indicate differences by diet effect; ** (*p* < 0.01) indicate differences by treatment effect; % (0.1 < *p* < 0.05), & (*p* < 0.05), and && (*p* < 0.01) indicate differences by photoperiod effect. ns, indicates no significance; STD, indicates standard diet-fed rats; CAF, indicates cafeteria diet-fed rats; VH, indicates rats administered vehicle; GSPE, indicates rats were administered with grape seed proanthocyanidin extract at 25 mg/kg b.w.; L6, indicates short photoperiod with 6 h light per day; L18, indicates long photoperiod with 18 h light per day.

**Table 2:** Nucleotide sequences of the primers used for the RT-qPCR.

Gene	Accession Number (NCBI)	Forward Primer (5′ to 3′)	Reverse Primer (5′ to 3′)
*Nrf2*	NM_001399173.1	ACATTTCAGTCGCTTGCCCT	TCCTGCCAAACTTGCTCCAT
*Keap1*	NM_057152.2	TGGGCGTGGCAGTGCTCAAC	GCCCATCGTAGCCTCCTGCG
*Sod1*	NM_017050.1	GGTGGTCCACGAGAAACAAG	CAATCACACCACAAGCCAAG
*Sod2*	NM_017051.2	AAGGAGCAAGGTCGCTTACA	ACACATCAATCCCCAGCAGT
*GPx1*	NM_030826.4	TGCAATCAGTTCGGACATC	CACCTCGCACTTCTCAAACA
*GSR*	NM_053906.2	ATCAAGGAGAAGCGGGATG	GCGTAGCCGTGGATGACT
*Nqo1*	NM_017000.3	GGGGACATGAACGTCATTCTCT	AGTGGTGACTCCTCCCAGACAG
*Sesn2* *Ppia*	NM_001109358.2 NM_017101.1	TACCTTAGCAGCTTCTGGCG TCAAACACAAATGGTTCCCAGT	AGGTAAGAACACTGGTGGCG ATTCCTGGACCCAAAACGCT

## Data Availability

The data presented in this study are available upon request from the corresponding author. The data are not publicly available because of a lack of a platform to publish them.

## Supplementary Materials

The supporting information can be downloaded at: https://www.mdpi.com/article/10.3390/ijms242317057/s1.

## Abbreviations

ARE: antioxidant response elements; b.w.: body weight; BCA: Pierce bicinchoninic acid; CAF: cafeteria diet; EGCG: (−)-epigallocatechin-3-gallate; GPx1: glutathione peroxidase; GSH: glutathione; GSPE: grape seed proanthocyanidin extract; GSR: glutathione-disulfide reductase; HO-1: heme-oxygenase 1; L12: standard photoperiod (12 h light/day); L18: long photoperiod (18 h light/day); L6: short photoperiod (6 h light/day); MetS: metabolic syndrome; NAFLD: nonalcoholic fatty liver disease; NEFAs: nonesterified fatty acids; *Nfe2l2*: nuclear factor erythroid-derived 2-like 2; NQO1: NAD(P)H quinone oxidoreductase 1; PCA: principal component analysis; PMSF: phenylmethylsulfonyl fluoride; ROS: reactive oxygen species; SCN: suprachiasmatic nucleus; SESN2: sestrin-2; SOD: superoxide dismutase; sPLS-DA: sparse partial least squares discriminant analysis; STD: standard diet; TAGs: triglycerides; TC: total cholesterol; VH: vehicle; ZT: *Zeitgeber*.
