# Supplementary material for: Abrupt Photoperiod Changes Differentially Modulate Hepatic Antioxidant Response in Healthy and Obese Rats: Effects of Grape Seed Proanthocyanidin Extract (GSPE)
Source: Int J Mol Sci. 2023 Dec 2;24(23):17057. doi: 10.3390/ijms242317057 (PMC10707189; doi:10.3390/ijms242317057)
Supplement: Supplementary file 1 [file ijms-24-17057-s001.zip › ijms-2725894-supplementary.pdf]

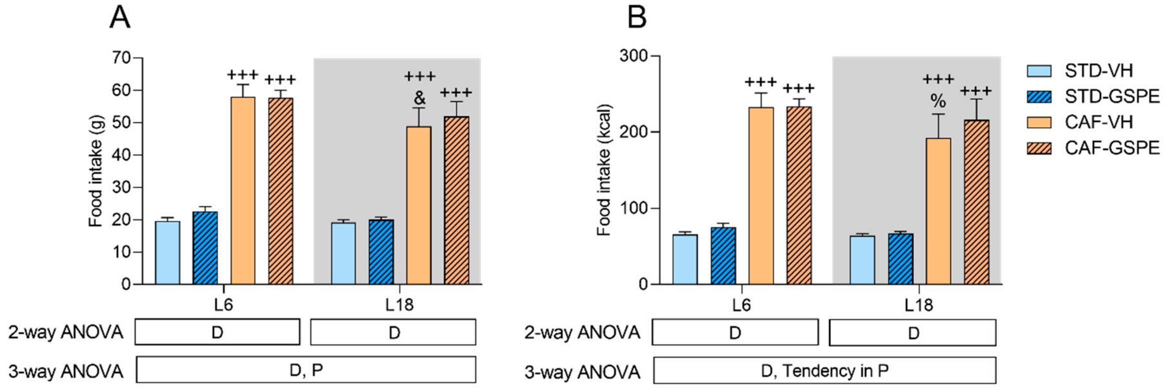

Figure S1: Food intake during the 7th week expressed in (**A**) grams and in (**B**) kcal. Values are expressed as the mean ± S.E.M. ($n = 4 - 6$) for L6 and L18 conditions. Statistical analyses were performed using 2 and 3-way ANOVA. The letters D and P refer to diet (STD *vs*. CAF) and photoperiod (L6 *vs*. L18) effect, respectively. LSD post-hoc test was used to compare between groups: +++ ($p < 0.001$) indicate differences by diet effect; % ($0.1 < p < 0.5$), & ($p < 0.05$) indicate differences by photoperiod effect. STD indicates Standard diet-fed rats; CAF indicates Cafeteria diet-fed rats; VH indicates rats administered vehicle; GSPE indicates rats were administered with grape seed proanthocyanidin extract at 25 mg/kg b.w.; L6 indicates short photoperiod with 6 h light per day and L18 indicates long photoperiod with 18 h light per day.

Table S1: Serum biochemical parameters.

Parameter	L6				L6 ANOVA	L18				L18 ANOVA	3-way ANOVA
	STD-VH	STD-GSPE	CAF-VH	CAF-GSPE		STD-VH	STD-GSPE	CAF-VH	CAF-GSPE		
Cholesterol (mM)	2.01 ± 0.13	2.01 ± 0.08	2.35 ± 0.2	2.4 ± 0.24	Tendency in D	2.2 ± 0.1	2 ± 0.13	3.5 ± 0.33$^{+++\&\&\&}$	2.47 ± 0.11**	D, T, Tendency in DxT	D, T, P, TxP, Tendency in DxP
Triglycerides (mM)	0.75 ± 0.01	0.83 ± 0.06	1.6 ± 0.15^{+++}	1.66 ± 0.11^{+++}	D	0.82 ± 0.12	0.81 ± 0.07	2.41 ± 0.26$^{+++\&\&\&}$	1.78 ± 0.12^{+++**}	D, Tendency in T, DxT	D, P, DxP, Tendency in TxP
NEFAs (mM)	648.93 ± 82.57	577.83 ± 116.43	685.1 ± 23.16	783.34 ± 48.18	ns	613.13 ± 108.45	594.68 ± 85.01	804.25 ± 87.4$^{\$}$	942.64 ± 105.01^{++}	D	D, Tendency in DxP
Glucose (mM)	7.57 ± 0.12	7.52 ± 0.18	8.88 ± 0.15^{++}	8.92 ± 0.33^{++}	D	7.82 ± 0.21	7.5 ± 0.23	9.48 ± 0.17^{+++}	9.55 ± 0.63^{+++}	D	D, Tendency in P
Insulin (mU/L)	0.18 ± 0.02	0.17 ± 0.02	0.31 ± 0.04^{++}	0.19 ± 0.01**	D, T, Tendency in DxT	0.22 ± 0.03	0.18 ± 0.01	0.2 ± 0.01$^{\&\&}$	0.24 ± 0.05$^{\$}$	ns	D, DxTxP, Tendency in T, TxP

Values are expressed as the mean ± S.E.M. (*n = 5 - 6*) for L6 and L18 conditions. Statistical analyses were performed using 2 and 3-way ANOVA. The letters D, T, and P refer to diet (STD *vs*. CAF), treatment (VH *vs*. GSPE), and photoperiod (L6 *vs.* L18) effect, respectively. LSD post-hoc test was used to compare between groups: $ (0.1 < *p* < 0.5), ++ (*p* < 0.01), and +++ (*p* < 0.001) indicate differences by diet effect; ** (*p* < 0.01) indicates differences by treatment effect; && (*p* < 0.01), &&& (*p* < 0.001) indicate differences by photoperiod effect. ns indicates no significance; STD indicates Standard diet-fed rats; CAF indicates Cafeteria diet-fed rats; VH indicates rats administered vehicle; GSPE indicates rats were administered with grape seed proanthocyanidin extract at 25 mg/kg b.w.; L6 indicates short photoperiod with 6 h light per day and L18 indicates long photoperiod with 18 h light per day.

Table S2: Liver antioxidant-related metabolites in L6 and L18 conditions, expressed in arbitrary units.

Metabolites	L6				L6	L18				L18
	STD-VH	STD-GSPE	CAF-VH	CAF-GSPE	*p*-value	STD-VH	STD-GSPE	CAF-VH	CAF-GSPE	*p*-value
Adenosine	0.12 (0.09 - 0.14)	0.17 (0.12 - 0.18)	0.2 (0.15 - 0.27)$	0.17 (0.15 - 0.17)	0.700	0.2 (0.12 - 0.29)	0.16 (0.13 - 0.22)	0.14 (0.1 - 0.22)	0.14 (0.09 - 0.18)	0.743
Adenosine-5-monophosphate Adenosine-5-diphosphate Adenosine-5-triphosphate	0.67 (0.57 - 0.71)	0.55 (0.35 - 0.97)	0.61 (0.56 - 0.64)	0.57 (0.55 - 0.58)	0.373	0.58 (0.51 - 0.97)	0.57 (0.52 - 0.73)	0.74 (0.55 - 0.89)	0.64 (0.54 - 0.74)	0.916
α-ketoglutaric acid	0.04 (0.03 - 0.05)	0.05 (0.04 - 0.06)	0.05 (0.04 - 0.05)	0.05 (0.04 - 0.07)	0.588	0.04 (0.03 - 0.04)	0.04 (0.04 - 0.05)	0.05 (0.05 - 0.06)++%	0.04 (0.03 - 0.05)	0.042
Aspartic acid	0.82 (0.8 - 0.92)	0.89 (0.78 - 1)	0.92 (0.81 - 1.15)	1.04 (0.98 - 1.12)	0.328	0.8 (0.73 - 0.9)	0.84 (0.77 - 0.86)	1.04 (0.83 - 1.31)	1.07 (0.89 - 1.28)	0.855
Citric acid	0.39 (0.33 - 0.51)	0.34 (0.3 - 0.35)	0.36 (0.25 - 0.48)	0.41 (0.32 - 0.74)	0.483	0.28 (0.18 - 0.33)	0.25 (0.24 - 0.33)	0.32 (0.26 - 0.34)	0.32 (0.3 - 0.41)	0.617
Fumaric acid	14.2 (12.87 - 15.7)	16.33 (14.62 - 17.53)	11.89 (10.75 - 14.28)	12.46 (10.72 - 16.07)	0.374	12.6 (9.95 - 13.91)	12.26 (11.17 - 13.24)	13.5 (12.69 - 14.41)	12.9 (9.85 - 14.51)	0.767
Glucose 6-phosphate	0.06 (0.04 - 0.08)	0.04 (0.03 - 0.05)	0.05 (0.05 - 0.06)	0.04 (0.04 - 0.05)	0.352	0.04 (0.04 - 0.05)	0.04 (0.03 - 0.05)	0.04 (0.04 - 0.06)	0.04 (0.03 - 0.05)	0.406
Glutamic acid	4.23 (2.84 - 4.45)	4.45 (3.88 - 5.26)	4.76 (4.27 - 4.93)	4.43 (4.38 - 4.5)	0.355	4.27 (3.16 - 5.2)	3.46 (2.88 - 4.07)	6.46 (5.75 - 7.37)+%	5.89 (4.88 - 6.92)+	0.168
Glycine	2.59 (2.49 - 2.79)	2.65 (2.57 - 3.04)	3.39 (2.79 - 3.57)$	2.89 (2.86 - 3.02)	0.230	2.76 (2.22 - 3.11)	2.38 (2.25 - 2.55)	3.79 (3.01 - 3.88)$	2.77 (2.65 - 3.01)$	0.395
Hydroxyphenyllactic acid	0.35 (0.32 - 0.47)	0.41 (0.33 - 0.47)	0.32 (0.23 - 0.34)	0.26 (0.1 - 0.31)+	0.235	0.5 (0.41 - 0.52)	0.43 (0.38 - 0.45)	0.22 (0.15 - 0.25)+	0.32 (0.25 - 0.32)+	0.234
Inosine	0.25 (0.19 - 0.54)	0.34 (0.27 - 0.43)	0.35 (0.28 - 0.48)	0.28 (0.26 - 0.32)	0.678	0.3 (0.25 - 0.34)	0.28 (0.21 - 0.33)	0.37 (0.29 - 0.47)	0.3 (0.25 - 0.32)	0.202
Inosine 5-monophosphate	0.05 (0.05 - 0.05)	0.04 (0.02 - 0.06)	0.06 (0.06 - 0.07)+	0.05 (0.05 - 0.06)*	0.066	0.04 (0.04 - 0.06)	0.04 (0.04 - 0.05)	0.08 (0.06 - 0.1)+	0.06 (0.06 - 0.07)$	0.490
Leucine	0.86 (0.71 - 0.99)	0.97 (0.84 - 1.08)	0.87 (0.77 - 1.03)	0.78 (0.71 - 0.85)	0.911	1.11 (0.96 - 1.14)	0.88 (0.84 - 0.91)	1.05 (0.7 - 1.13)$	0.78 (0.71 - 0.9)	0.049
Malic acid	4.8 (4.43 - 5.15)	4.6 (4.15 - 6.11)	3.66 (3.26 - 4.13)+	4.37 (4.02 - 4.97)	0.127	4.07 (3.01 - 5.18)	4.01 (3.59 - 4.36)	4.46 (3.53 - 5.38)	4.53 (3.86 - 4.76)	0.701
Methionine	1.12 (1.11 - 1.16)	1.17 (1.11 - 1.2)	1.05 (1.03 - 1.14)	1.08 (1.05 - 1.14)+	0.224	1.16 (1.09 - 1.17)	1.15 (1 - 1.16)	1.11 (1.04 - 1.12)	1.08 (1.05 - 1.09)	0.315
myo-Inositol	0.31 (0.26 - 0.38)	0.33 (0.31 - 0.39)	0.3 (0.25 - 0.31)	0.25 (0.23 - 0.28)	0.598	0.35 (0.26 - 0.43)	0.3 (0.29 - 0.35)	0.32 (0.23 - 0.35)	0.27 (0.25 - 0.34)	0.528
Nicotinamide	0.53 (0.49 - 0.86)	0.71 (0.64 - 0.75)	0.72 (0.63 - 0.87)	0.69 (0.6 - 0.86)	0.683	0.69 (0.56 - 0.93)	0.64 (0.6 - 0.82)	0.69 (0.55 - 0.91)	0.55 (0.54 - 0.59)	0.393
Phenylalanine	0.37 (0.29 - 0.42)	0.37 (0.37 - 0.41)	0.37 (0.34 - 0.44)	0.35 (0.32 - 0.36)	0.931	0.45 (0.41 - 0.47)	0.35 (0.33 - 0.38)#	0.46 (0.31 - 0.49)	0.32 (0.31 - 0.4)	0.046
Phosphoric acid	44.73 (41.18 - 46.16)	44.9 (43.59 - 46.49)	46.67 (46.63 - 48.13)	45.77 (43.95 - 47.4)	0.732	44.5 (40.51 - 47.46)	44.91 (43.51 - 45.23)	47.33 (42.1 - 49.51)	43.16 (41.16 - 44.78)	0.312
Proline	2.33 (1.82 - 2.55)	2.47 (2.12 - 2.8)	2.79 (2.46 - 3.08)	2.61 (2.29 - 2.64)	0.546	2.88 (2.57 - 2.98)	2.43 (2.16 - 2.96)	3.59 (2.41 - 3.88)	2.79 (2.52 - 3.38)	0.963
Pyruvic acid	0.55 (0.51 - 0.63)	0.59 (0.47 - 0.64)	0.67 (0.48 - 0.78)	0.64 (0.55 - 0.7)	0.543	0.77 (0.67 - 0.91)%	0.73 (0.67 - 0.81)	0.92 (0.54 - 1.11)	0.9 (0.6 - 1.06)	0.916
Ribose-5-phosphate	0.04 (0.03 - 0.04)	0.04 (0.03 - 0.04)	0.04 (0.04 - 0.04)	0.04 (0.04 - 0.04)	0.779	0.05 (0.04 - 0.05)%	0.04 (0.04 - 0.04)	0.04 (0.03 - 0.05)	0.03 (0.03 - 0.05)	0.531
Sarcosine	0.03 (0.02 - 0.03)	0.03 (0.02 - 0.03)	0.01 (0.01 - 0.02)+	0.01 (0.01 - 0.02)+	0.009	0.04 (0.03 - 0.04)&	0.03 (0.03 - 0.04)&	0.01 (0.01 - 0.02)++	0.01 (0.01 - 0.02)++	0.000
Serine	0.81 (0.56 - 0.99)	1.03 (0.98 - 1.03)	2.39 (1.94 - 3.22)++	2.33 (1.88 - 2.58)$	0.014	0.64 (0.51 - 0.97)	0.53 (0.45 - 0.83)&	3.7 (3.34 - 3.81)++&	2.09 (1.49 - 2.79)++**	0.181
Stearic acid	15.41 (13.05 - 18.6)	14.15 (13.19 - 14.78)	21.19 (16.79 - 22.64)	19.49 (17.53 - 24.53)+	0.555	13.52 (12.45 - 16.95)	13.77 (12.32 - 14.38)	17.42 (15.9 - 19.18)	17.73 (15.64 - 21.45)$	0.117

Taurine	4.57 (3.45 - 6.41)	3.5 (2.55 - 3.73)	3.13 (1.81 - 3.88)	4.53 (1.48 - 6.44)	0.507	5.84 (2.69 - 6.95)	7.5 (6.23 - 7.81)&	1.62 (1.14 - 3.05)+	3.08 (2.75 - 4.84)$	0.364
Threonine	2.91 (2.69 - 3)	2.75 (2.51 - 2.91)	2.7 (2.59 - 2.83)	2.83 (2.69 - 2.97)	0.248	2.6 (2.53 - 2.66)%	2.6 (2.51 - 2.68)	2.75 (2.63 - 2.85)$	2.72 (2.71 - 2.81)$	0.134

Values are expressed as the median (interquartile range, IQR) in arbitrary units. Statistical analyses were performed using Kruskal-Wallis' test. Mann-Whitney U test was used to compare between groups: $ (0.1 < *p* < 0.5), + (*p* < 0.05), and ++ (*p* < 0.01) indicate differences by diet effect; # (0.1 < *p* < 0.5), * (*p* < 0.05), and ** (*p* < 0.01) indicates differences by treatment effect; % (0.1 < *p* < 0.5) and & (*p* < 0.05) indicate differences by photoperiod effect. STD indicates Standard diet-fed rats; CAF indicates Cafeteria diet-fed rats; VH indicates rats administered vehicle; GSPE indicates rats administered 25 mg/kg BW grape seed proanthocyanidin extract; L6 indicates short photoperiod with 6 h light per day and L18 indicates long photoperiod with 18 h light per day.
